# A Case of Peritoneal Adhesions of Head of Colon Simulating Appendicitis

**Published:** 1895-03

**Authors:** A. F. Harrington

**Affiliations:** Atlanta, Ga.; No. 308 Equitable


### A CASE OF PERITONEAL ADHESIONS OF HEAD OF COLON, SIMULATING APPENDICITIS.

BY A. F. HARRINGTON, A. B., M. D., ATLANTA, GA.

A negro man of a large and muscular build, age 26 years, came to my office, on October 20th, 1894, suffering with pain in the right inguinal region, directly above the appendix vermiformis. The advent of this he dated from a strain received several days previous to the time I saw him. There was considerable tenderness on pressure, but no tumor. Constipation and a slight elevation of temperature existed.

These symptoms rapidly abated under treatment, which consisted of absolute rest in bed, a nightly laxative dose of fluid extract cascara sagrada and fluid diet. In the course of ten days the man, considering himself well, went back to his work, which served to return his pain immediately. The former treatment together with an occasional hypodermic injection of morphine did nothing more than palliate his suffering. The pain and tenderness grew more acute each day, a small tumor could with difficulty be mapped out. Temperature now ranged between 100° and 103° Fahr. Right leg slightly drawn up, but could be straightened with ease.

Pronounced dullness on percussion and an imaginary fluctuation obtained, led me to think that conservative treatment was no longer advisable. A consultation decided in favor of an operation, with the idea that our patient was suffering with appendicitis.

This operation was performed on December 5th. A three-inch incision was made over appendex, in the line suggested by Dr. Wyeth. When abdominal cavity was reached, instead of finding an appendicitis, we found the head of colon firmly bound down by peritoneal adhesions and surrounded by a mass of enlarged and hardened mesenteric glands. The appendix showed no signs of previous inflammation, though its mesentery was firmly adherent to colon. All these adhesions were dissected loose with my fingers and the colon freed. The incision was now closed without drainage. Now what appears remarkable to me is that all pain and fever ceased immediately, and on tenth day after operation, wound was completely healed. Not even a reactionary temperature was experienced. Patient dismissed on December 20th.

Was again called on January 15th to find that a large hemorrhage from the bowels had occurred, due, I thought, to a tuberculous ulcer of the colon opening a blood vessel.

Our patient succumbed to this tuberculosis of the intestines on January 29th.

I report this case as being one unique in its symptoms, and for the surprising result of giving comfort to a patient by an operation which was apparently worse than useless. I offer no explanation for the condition of the head of colon except the probability of it being a tuberculous deposit.

*No. 308 Equitable.*

